# Effect of microclimatic temperatures on the development period of 3 rice planthopper species (Hemiptera: Delphacidae): a phenology model based on field observations

**DOI:** 10.1093/ee/nvae005

**Published:** 2024-01-22

**Authors:** Ryota Mochizuki, Toshihisa Yashiro, Sachiyo Sanada-Morimura, Atsushi Maruyama

**Affiliations:** Kyushu Okinawa Agricultural Research Center, National Agriculture and Food Research Organization (NARO), 2421 Suya, Koshi, Kumamoto 861-1192, Japan; Koshi Campus, Institute for Plant Protection, National Agriculture and Food Research Organization (NARO), 2421 Suya, Koshi, Kumamoto 861-1192, Japan; Koshi Campus, Institute for Plant Protection, National Agriculture and Food Research Organization (NARO), 2421 Suya, Koshi, Kumamoto 861-1192, Japan; Institute of Agro-Environmental Sciences, National Agriculture and Food Research Organization (NARO), 3-1-3 Kannondai, Tsukuba 305-8604, Japan

**Keywords:** microclimate, phenology model, rice planthopper, canopy, microhabitat

## Abstract

Most pest phenology models are temperature dependent. Generally, the air temperature at reference height is used to predict pest development, but the air temperature varies between inside and outside the crop canopy, where pests reside. Here, we sampled 3 rice planthopper species—*Nilaparvata lugens* (Stål), *Sogatella furcifera* (Horváth), and *Laodelphax striatellus* (Fallén)—and micrometeorological observations in paddy fields to analyze how thermal environments inside the canopy affect pest development. Seasonal variations in the population density of these species were surveyed in 3 experimental fields with 2 water temperature plots (normal and low-water temperature plots). The development periods of the 3 species were predicted individually based on pest phenology models using temperatures recorded at 6 heights (0.0–2.0 m). We calculated the root mean square error (RMSE) values from the predicted and observed development periods for each rice planthopper. The development prediction using the temperature inside the canopy was more accurate than that utilizing the temperature at the reference height (2.0 m). In the low-water temperature plot, the RMSE value for *N. lugens*, *S. furcifera*, and *L. striatellus* was 6.4, 5.6, and 4.1 when using the temperature at the reference height (2.0 m), respectively, and 2.8, 3.8, and 2.9 when employing the temperature inside the canopy at 0.25 m, respectively. The development prediction utilizing the air temperature at the bottom (0.25 m) of canopy, where *N. lugens* resides, was most effective for *N. lugens* among the 3 species. These findings suggest the importance of utilizing microhabitat-based temperatures to predict pest development.

## Introduction

Pest phenology models have been developed to predict the development stages, thereby serving as a tool for determining the optimal timing of pesticide application (Utida 1957, Damos and Savopoulou-Soultani 2012, Tonnang et al. 2017). These models, aimed at forecasting the seasonal prevalence of pest occurrences, have been extensively researched for various insect species, including lepidopteran and hemipteran insects (Davidson 1944, Hilbert and Logan 1983, Honek and Kocourek 1990, Kiritani 2012). Generally, phenology models rely solely on temperature as the environmental variable, and they are developed in laboratories with strictly controlled homogeneous temperatures (Noda 1989, Honek and Kocourek 1990, Kontodimas et al. 2004, Nietschke et al. 2007, Kiritani 2012, Park et al. 2013, Vattikuti et al. 2019, Yang et al. 2021). Therefore, utilizing the temperature corresponding to the microhabitat where pests reside is preferred for predicting pest development using the phenology model in the field environment.

Air temperature at a reference height (1.5–2.0 m) near a target field is most commonly used for pest development prediction. However, pests typically inhabit the canopies of cultivated crops, and environmental conditions, including temperature, vary between inside crop canopies and typical reference heights (Takai et al. 2010, Yoshimoto et al. 2011, 2022, Fukuoka et al. 2012). Previous research has emphasized the importance of using microclimate data to predict diseases and pest damage (Hatfield 1982, Kuwagata et al. 2019). Specifically, several studies have assessed the influence of microclimate on disease occurrence within crop canopies through field surveys (Pangga et al. 2013, Cheng et al. 2015, Haider et al. 2017). However, research focusing on pest development within the canopy is limited, and existing studies have heavily relied on simulation-based validation approaches rather than field surveys (Davidson et al. 1990, Saudreau et al. 2013). Some studies have found that using air temperature inside the canopy improved the accuracy of predicting potato moth development (Rebaudo et al. 2016, Faye et al. 2017). Studies using microclimatic temperature as an environmental variable for predicting pest development have been restricted to dry-field crops and associated pests. In paddy rice, the air temperature inside the canopy is susceptible to flooding, drained conditions, and water temperature (Miyasaka et al. 2011, Tsujimoto et al. 2021, Sun et al. 2022, Maruyama et al. 2023). Consequently, the temperature difference between inside and outside the canopy may be more pronounced in paddy fields than in dry-field crops. To date, no studies have examined temperature inside the canopy and pest development in paddy field crops. Therefore, in this study, we investigated the effect of temperature inside the canopy on pest development in paddy rice.

*Nilaparvata lugens* (Stål), *Sogatella furcifera* (Horváth), and *Laodelphax striatellus* (Fallén), rice planthopper species, are major paddy rice pests in Asia. They considerably decrease paddy rice yield and quality through direct phloem-sap feeding and pathogenic virus transmission (Bottrell and Schoenly 2012, Lee et al. 2013, Horgan et al. 2015, Li et al. 2015, Matsumura 2017, Jiang et al. 2018, Yang et al. 2021). In temperate regions of East Asia, including Japan, *N. lugens* and *S. furcifera* are unable to overwinter and establish themselves after long-distance migration from overwintering areas, such as southern China and northern Vietnam (Matsumura 2017). In contrast, *L. striatellus* is an indigenous insect that can overwinter in Japan and can invade rice paddies through migration from nearby weeds rather than from overseas flights (Shiba et al. 2018). Existing methods to predict the development of the 3 species have utilized temperatures observed at reference heights, as with other insects (Okuda et al. 2019, Tanaka et al. 2019). However, as *N. lugens*, *S. furcifera*, and *L. striatellus* reside within the paddy rice canopy, they experience temperatures different from those at reference heights. Notably, the 3 rice planthopper species exhibit variations in microhabitat preferences within the canopy; *N. lugens* tends to inhabit the lower parts of rice plants, whereas *S. furcifera* and *L. striatellus* do not exhibit the same behavior (Takai and Inou 1970, Noda 1987, Isichaikul and Ichikawa 1993, Matsumura 2017).

In this study, we examined the population density of the 3 rice planthopper species (*N. lugens*, *S. furcifera*, and *L. striatellus*) and conducted meteorological observations inside and outside the canopy. We recorded temperatures at various heights in 3 experimental fields with different water temperature plots to gather a comprehensive dataset of canopy temperatures. Based on the population dynamics of these 3 species within the experimental plots, the relationships between the development prediction in rice planthoppers and the microclimate within the canopy were discussed.

## Materials and Methods

### Experimental Site

In 2021, all experiments were conducted in 3 rice paddy fields (10 × 50 m) of the Kyushu Okinawa Agricultural Research Center, NARO, Suya, Koshi, Kumamoto, Japan (32°52ʹ26 ʹN, 130°44ʹ25ʹE) where the rice cultivar—*Oryza sativa* (variety, Nikomaru)—was transplanted on different dates. Growing season (transplanting to harvest) for the May, June, and July transplants was 25 May–6 October 2021; 21 June–28 October 2021; and 8 July–28 October 2021, respectively. The planting density was maintained at 16.7 plants per square meter (0.3 × 0.2 m), and chemical fertilizers (6.4 g/m each of nitrogen, phosphorus, and potassium) were applied. The only chemicals used were herbicides, applied 3 wk after transplantation.

In each paddy field, 2 plots (10 × 25 m) with different water temperatures (normal and low) were established to obtain broad-range temperature data inside the canopy and to ensure easier examination of the effects of the temperature inside the canopy on the development of the 3 planthopper species. Upstream plots created by continuous irrigation and paddy field partitions were defined as “low-water temperature” plots and downstream plots as “normal” plots (Miyasaka et al. 2011). The low-water temperature plots in this study were replicates of paddy fields in mountainous areas and those grown using groundwater. Water levels were adjusted to an average of 80 mm, and no mid-drainage was conducted.

### Meteorological Observation

Meteorological observations were conducted at the center of each plot throughout the rice growing season to minimize edge effects. Air temperatures inside and outside the canopy were observed using ultra-fine T-type thermocouples (φ 0.08 mm; OMEGA Engineering, Norwalk, CT, USA) to minimize the influence of radiation. The effect of solar radiation increases with sensor size (Bergman et al. 2020). Maruyama et al. (2020) reported a daily mean error of 0.24 °C when using sensors with 0.25-mm fine thermocouples. The sensor used in this study was 0.08 mm, indicating that the effect of solar radiation was even smaller than that reported in the previous studies. In addition, the effect of solar radiation was further diminished as the temperature was observed inside the canopy. Therefore, using the 0.08-mm sensor rendered the effect of solar radiation on temperature negligible. At 2.0-m height, the air temperature was measured above the ground using a naturally ventilated shelter (41303-5A, Young, MI, USA), whereas air temperatures inside the canopy were measured at 1.0, 0.75, 0.5, and 0.25 m above the ground without a shelter to eliminate the effect of shelter heating by radiation in the canopy where adequate ventilation was difficult. Water temperature was observed mid-depth at 3 points with T-type thermocouples (φ 0.65 mm, OMEGA Engineering); the water temperature averages were used during analysis. Meteorological observations were made every 10 s with data loggers (CR1000 and CR1000X, Campbell Scientific, Logan, UT, USA) and recorded as 10-min averages.

### Surveys of Rice Planthopper Species

In each experimental plot, *N. lugens*, *S. furcifera*, and *L. striatellus*, 3 major Asian pest species, were surveyed. The arrival time of *N. lugens* and *S. furcifera* in rice paddies was determined based on the date of capture of adults via net traps set at 10-m height (Kisimoto 1976, Otuka 2013). Net traps placed next to paddy fields were checked daily during the growing season of paddy rice. A total of 320 adult *N. lugens* male and female individuals (male–female ratio of 1:1) were released into each experimental plot on 30 July because there was no sufficient invasion to establish themselves in the experimental plots. After the first 2 wk of transplantation, the population density of rice planthoppers in paddy fields was surveyed twice a week using the standard sticky board method. In this method, a sticky board was placed horizontally on one side of a rice hill at 10 cm above the ground, and the rice hill was manually struck on the other side to dislodge the insects (Nagata and Masuda 1978). The survey was conducted in 3 replicates of 20 rice hills per experimental plot, and the survey data averages were used to calculate the population density of each species per plot. The development period of rice planthoppers, defined according to previous reports, is an indicator used to verify the accuracy of development prediction using temperature (Kuno 1968, Tanaka et al. 2019). Specifically, the development period was defined as the period between the date of first arrival of a species (including the date of release of *N. lugens*) and the peak of population density (or the percentage of adult population) after the predicted development period (occurring after 20–30 days of arrival in the case of rice planthoppers) or as the period between peak of population density (or the percentage of adult) and peak of population density (occurring after 20–30 days of population density peak). The percentage of adult population was calculated using adult and nymph population densities. In this study, only life stages after the middle-nymph stage were counted because early nymphs are difficult to distinguish among the 3 species.

### Phenology Model

A linear model was used to predict pest development (Uvarov 1931, Utida 1957, Damos and Savopoulou-Soultani 2012). Details of each parameter based on previous laboratory experiments are shown in Table 1. The models predicted the development of the 3 rice planthopper species at each of the 3 different stages (egg, nymph, and preoviposition [PO]). Rice planthoppers progressed to the next stage when the hourly integrated value of the developmental rate reached *R* = 1, and the predicted development period was defined as the period when all development stages were completed. The temperatures used in the phenology model were hourly averages of the observed values.

**Table 1.** Parameters for the linear models of *Nilaparvata lugens* (Stål), *Sogatella furcifera* (Horváth), and *Laodelphax striatellus* (Fallén)

Species	Stage	Linear model				References
		*T*_min_	*T*_upper_	*T*_stop_	*K*	
*N. lugens*	Egg	12.7	28.5	35.0	109.4	Noda (1989), Krishnaiah et al. (2005)
	Nymph	11.3	28.5	34.9	194.9	Noda (1989), Piyaphongkul et al. (2012), Horgan et al. (2020)
	PO	11.4	30.0	37.0	35.9	Noda (1989), Piyaphongkul et al. (2012), Horgan et al. (2020)
*S. furcifera*	Egg	12.6	28.5	37.8	78.0	Suenaga 1963), Noda (1989), Park et al. (2013)
	Nymph	11.2	28.5	35.0	178.9	Suenaga (1963), Noda (1989), Park et al. (2013)
	PO	10.7	30.0	37.8	65.2	Noda (1989), Horgan et al. (2020)
*L. striatellus*	Egg	11.7	29.0	40.0	114.0	Noda (1989), Hirae and Shiba (2016)
	Nymph	10.8	29.0	40.0	212.1	Noda (1989), Hirae and Shiba (2016)
	PO	11.2	29.0	40.0	54.3	Noda (1989), Hirae and Shiba (2016)

PO represents preoviposition. *T*_min_ is the lower threshold temperature for development, *K* is the thermal constant, *T*_upper_ is the upper threshold temperature limit for development, and *T*_stop_ is the temperature at which development stops or the survival rate is zero (i.e., the critical thermal maximum).

The linear model with effective accumulated temperature is the most commonly applied model; it describes the relationship between development rate and temperature as shown in Equation (1) (Noda 1989, Vattikuti et al. 2019):


$$\begin{array}{l} \begin{array}{l} R=1/P\;=(T-T_{min})/K\; \end{array} \end{array}$$ (1)

where *R* is the developmental rate, *P* is the developmental period, *T* is the temperature (the explanatory variable), *T*_min_ is the lower threshold temperature for development, and *K* is the thermal constant. In this study, the model was expressed with Eq. (2) when considering the effects of high temperatures (Okuda et al. 2019):


$$\begin{array}{l} \begin{array}{llll} & \begin{array}{ll} R=1/P\;= & \;(T-T_{min})/K,\;(T<T_{upper})\; \end{array}\; & \;\;\;\;=(T_{upper}-T_{min})/K,\;(T_{upper}\leq T<T_{stop})\; & \;\;\;\;=0,\;(Ts\leq T)\; \end{array} \end{array}$$ (2)

where *T*_upper_ is the upper-temperature threshold and *T*_stop_ is the temperature at which development stops and the survival rate is zero (i.e., the critical thermal maximum).

The threshold temperatures have been studied in various regions and found to be similar (Horgan et al. 2020). Specifically, Kiritani (2012) reported that numerous studies have examined *T*_min_. In this study, we used the values reported by Noda (1989) who examined all stages, including the PO stage. For each life stage of *N. lugens*, *T*_upper_ was the temperature reported by Horgan et al. (2020), whereas *T*_stop_ was derived from the temperatures reported by Krishnaiah et al. (2005) and Piyaphongkul et al. (2012). Similar previously reported values were referenced for *S. furcifera* and *L. striatellus* (Table 1).

### Data Analysis

The effects of water temperature differences on air temperature inside the canopy were confirmed using the Tukey honest significant difference (HSD) test (*P* ≤ 0.05). The prediction method using the air temperature inside the canopy and that using the air temperature at the reference height (2.0 m) was compared by calculating the root mean square error (RMSE) and mean absolute error (MAE) values between the predicted and observed development periods. Furthermore, the prediction methods were verified with skill scores based on RMSE. The RMSE skill score (RMSE_ss_) showed the proportion of RMSE improvement over a reference method. RMSE_ss_ was defined as RMSE_ss_ = 1 − (RMSE_h_/RMSE_ref_), where RMSE_h_ is the RMSE calculated using the temperature inside the canopy at a specific height and RMSE_ref_ is the RMSE calculated using the temperature at the reference height (2.0 m). All statistical analyses were performed using the “stats” package in R (v.4.1.2) (R Core Team 2021).

## Results

### Diurnal and Seasonal Variations in Air Temperature Inside the Canopy

**Fig. 1.**
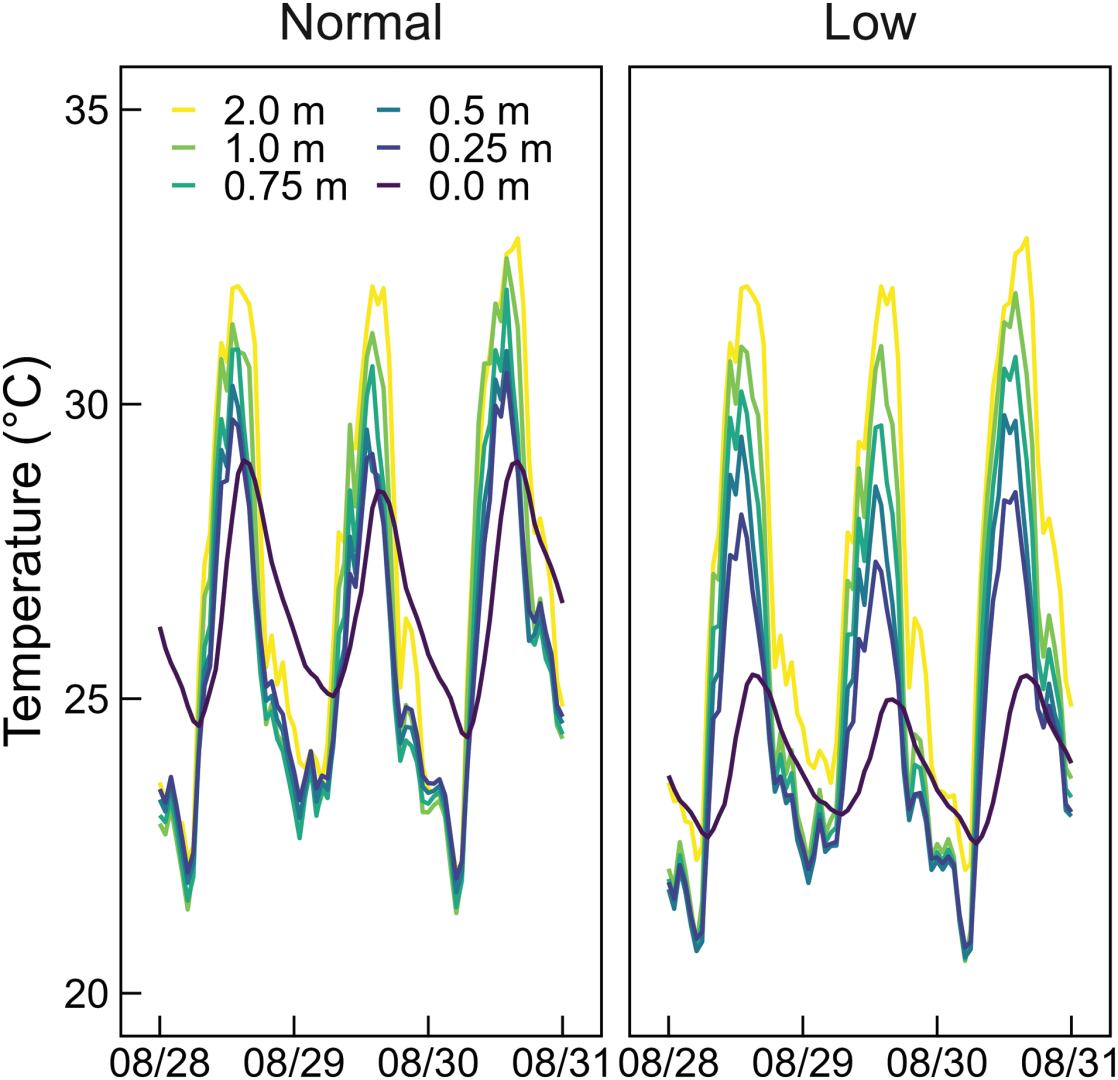
Diurnal variation in air and water (0.0 m) temperatures at different heights in normal and low-water temperature plots during the heading stage.

Figure 1 shows the comparison results of daily variations in air temperatures inside and outside the canopy and water temperatures on the heading date of paddy rice transplanted in June 2021 between normal and low-water temperature plots. The average height of paddy rice plants during this time was 1.1 m. Continuous irrigation and paddy field partitions resulted in 3–4 °C differences in water temperature during the day on the heading date. During the day, the air temperatures inside the canopy gradually increased from 0.25 to 2.0 m, with the lowest temperature observed at the water level (0.0 m). At night, the air temperatures inside the canopy were lower than the air temperature at the reference height (2.0 m) and the water temperature (0.0 m).

Table 2 shows the mean daily air temperatures inside and outside the canopy, water temperatures in each experimental plot, and the result of the Tukey-HSD test after the release of *N. lugens* (30 July–30 September 2021). The air temperature inside the canopy (0.25 m) in the normal and low-water temperature plots was 1.3 and 2.1 °C lower than the air temperature at the reference height (2.0 m), respectively. The results of the Tukey-HSD test revealed statistically significant differences between air temperatures inside (0.25–1.0 m) and above the canopy (2.0 m) in both plots. Additionally, there were also significant differences (*P* ≤ 0.05) in the air temperatures inside the canopy at several different heights.

**Table 2.** Daily temperatures (mean ± standard deviation) based on height in normal and low-water temperature plots during the growing season after the release of *Nilaparvata lugens* (Stål) (30 July–30 September 2021) in 3 fields and a result of 2-way ANOVA testing the effect of height and experimental plot

ANOVA	df	*F*-value	*P*-value
Height	5	60.41	<0.0001
Plot	1	85.30	<0.0001
Height × Plot	5	17.20	<0.0001
Height (m)	Average temperature (°C)		
	Normal plot		Low-water temperature plot
2.00	25.8 ± 1.81^a^		
1.00	25.1 ± 1.94^b^		24.8 ± 1.88^bc^
0.75	24.6 ± 1.82^bc^		24.3 ± 1.75^cd^
0.50	24.5 ± 1.74^cde^		23.9 ± 1.60^df^
0.25	24.4 ± 1.76^cde^		23.6 ± 1.46^f^
0.00	25.0 ± 2.08^be^		22.9 ± 0.98^g^

Among the observed heights, 0.25–2.00 m is the air temperature, and 0.00 m is the water temperature. Different letters indicate a significant difference according to Tukey’s honest significant difference (HSD) test (*P* ≤ 0.05).

### Rice Planthopper Development Period

The results of adult population peaks observed in the 3 experimental fields with 2 different water temperature plots based on the number of 3 rice planthopper species are shown in Table 3. The development periods were determined based on the results of adult peaks (population density and adult percentage). The starting dates of the development periods for *N. lugens* were 30 July, when their initial release was carried out, and 14 August, when an invasion from overseas was observed. *Sogatella furcifera* invaded multiple times, with major dates of 9 June, 9 July, and 14 August. *Laodelphax striatellus* is an indigenous insect that constantly invades paddies from nearby grasslands. Therefore, the dates of the first observation of *L. striatellus* or those of the adult peaks (population density and adult percentage) were used as the starting dates.

**Table 3.** Date of adult peaks based on the population dynamics of rice planthoppers—*Nilaparvata lugens* (Stål), *Sogatella furcifera* (Horváth), and *Laodelphax striatellus* (Fallén)—in normal and low-water temperature plots

Species	Transplant	Plot	Date			
			Start	Peak 1	Peak 2	Peak 3
*N. lugens*	May	Normal	30 July 2021	27 Aug 2021	28 Sept 2021	
			14 Aug 2021	7 Sept 2021	5 Oct 2021	
		Low	30 July 2021	27 Aug 2021		
			14 Aug 2021	14 Sept 2021		
	June	Normal	30 July 2021	27 Aug 2021		
			14 Aug 2021	14 Sept 2021		
		Low	30 July 2021	31 Aug 2021	5 Oct 2021	
			14 Aug 2021	14 Sept 2021		
	July	Normal	30 July 2021	24 Aug 2021	21 Sept 2021	
			14 Aug 2021	14 Sept 2021	19 Oct 2021	
		Low	30 July 2021	27 Aug 2021		
			14 Aug 2021	10 Sept 2021	15 Oct 2021	
			3 Sept 2021	9 Oct 2021		
*S. furcifera*	May	Normal	9 July 2021	6 Aug 2021	31 Aug 2021	
			14 Aug 2021	10 Sept 2021		
		Low	9 June 2021	6 July 2021	3 Aug 2021	
			14 Aug 2021	7 Sept 2021		
	June	Normal	9 July 2021	3 Aug 2021	3 Sept 2021	1 Oct 2021
			14 Aug 2021	10 Sept 2021	9 Oct 2021	
		Low	9 July 2021	3 Aug 2021	3 Sept 2021	
			20 Aug 2021	17 Sept 2021		
	July	Normal	3 Aug 2021	31 Aug 2021	28 Sept 2021	
			14 Aug 2021	14 Sept 2021	12 Oct 2021	
		Low	3 Aug 2021	3 Sept 2021	28 Sept 2021	
			14 Aug 2021	14 Sept 2021	12 Oct 2021	
*L. striatellus*	May	Normal	18 June 2021	13 July 2021	6 Aug 2021	27 Aug 2021
			13 Aug 2021	10 Sept 2021		
		Low	25 June 2021	20 July 2021		
			13 Aug 2021	10 Sept 2021		
	June	Normal	9 July 2021	6 Aug 2021	7 Sept 2021	15 Oct 2021
		Low	9 July 2021	6 Aug 2021	10 Sept 2021	
			20 July 2021	17 Aug 2021	17 Sept 2021	
	July	Normal	3 Aug 2021	31 Aug 2021		
			17 Aug 2021	17 Sept 2021		
		Low	3 Aug 2021	31 Aug 2021		
			20 Aug 2021	14 Sept 2021		

The average development periods observed in the normal and low-water temperature plots were 29.11 and 31.44 days for *N. lugens*, 27.92 and 27.80 days for *S. furcifera*, and 28.33 and 28.50 days for *L. Striatellus*, respectively. There were no statistically significant differences (*P* > 0.05) in the development periods between the plots for any species.

### Prediction of Rice Planthopper Development Period

**Fig. 2.**
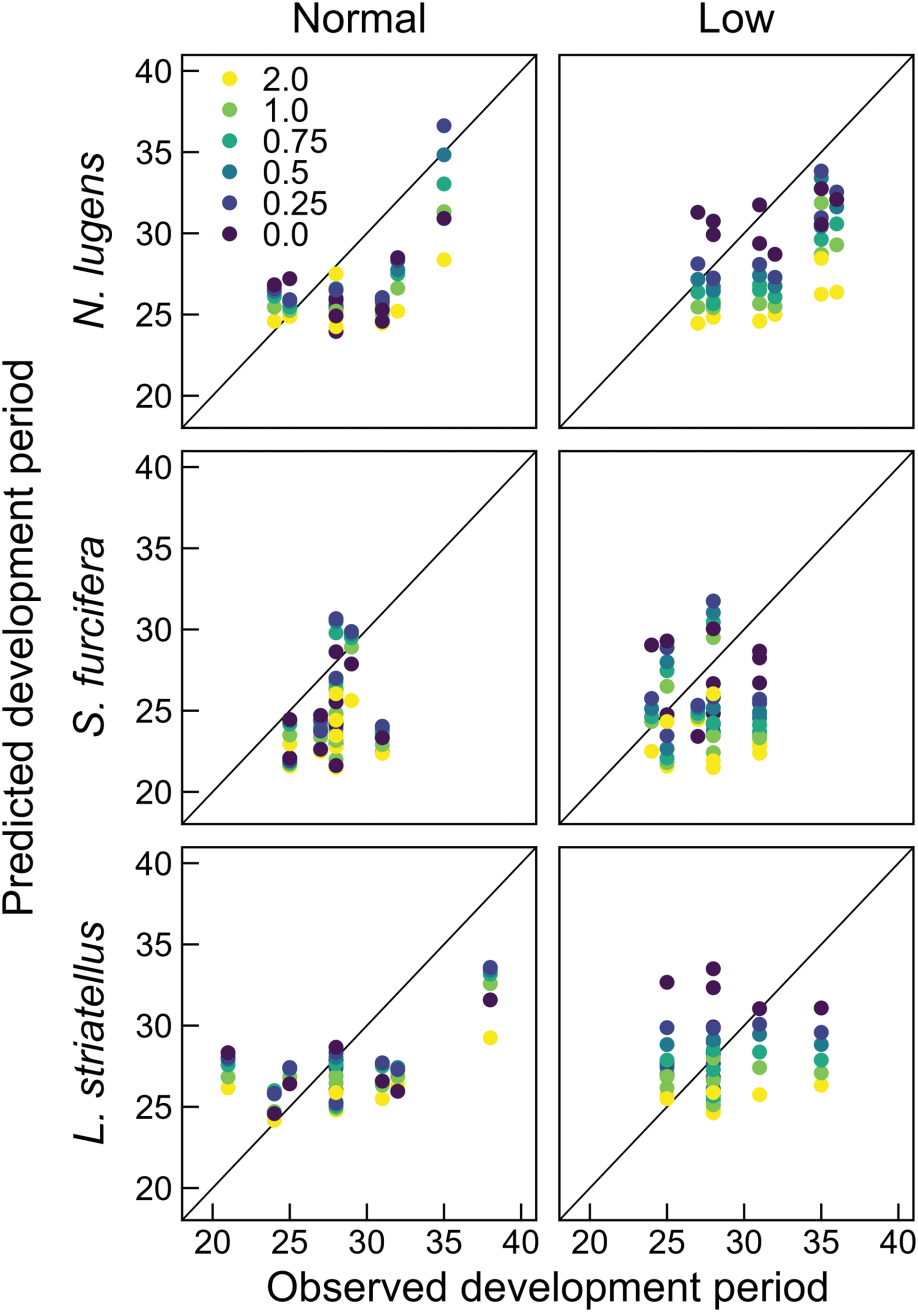
Relationships between the observed and predicted development periods (in days) of rice planthoppers based on height. The results of the normal and low-water temperature plots for *Nilaparvata lugens* (Stål) (*n* = 10, 9), *Sogatella furcifera* (Horváth) (*n* = 12, 10), and *Laodelphax striatellus* (Fallén) (*n* = 9, 8) are shown.

Figure 2 shows the relationship between the development periods predicted using air temperatures inside and outside the canopy or water temperatures and the observed development periods obtained from the population dynamics of the 3 rice planthopper species. For all 3 species, the development periods predicted using air temperature at the reference height (2.0 m) tended to be shorter than the observed development periods. The RMSE values for the 3 rice planthopper species were smaller when calculated using air temperatures inside the canopy (0.25–0.75 m) than when calculated using air temperatures at the reference height (2.0 m) (Table 4). In the normal plots, the RMSE minimum value for *N. lugens*, *S. furcifera*, and *L. striatellus* was 3.10 at 0.5 m height, 3.88 at 0.25–0.5 m height, and 3.61 at 0.5 m height, respectively. Similar tendencies were observed for the MAE values. In the low-water temperature plots, the RMSE and MAE minimum values for *N. lugens*, *S. furcifera*, and *L. striatellus* were observed at 0.25, 0.0, and 0.25 m heights, respectively. The RMSE_ss_ for *N. lugens* was 0.31 and 0.56 in the normal and low-water temperature plots when calculated using air temperature inside the canopy at 0.25 m height; the scores for *S. furcifera* and *L. striatellus* were 0.24 and 0.31, and 0.20 and 0.29, respectively.

**Table 4.** RMSE and MAE values were calculated using the observed and predicted development periods for the 3 rice planthopper species: *Nilaparvata lugens* (Stål), *Sogatella furcifera* (Horváth), and *Laodelphax striatellus* (Fallén). The development periods were predicted based on air temperature at each height in normal and low-water temperature plots

Species	Height (m)	RMSE (day)		MAE (day)	
		Normal	Low	Normal	Low
*N. lugens*	2.00	4.55	6.40	3.75	5.95
*n* = 10, 9	1.00	3.84	4.71	3.38	4.31
	0.75	3.25	4.00	2.82	3.57
	0.50	3.10	3.33	2.61	2.88
	0.25	3.12	2.81	2.78	2.43
	0.00	4.02	3.05	3.77	2.81
*S. furcifera*	2.00	5.11	5.57	4.69	4.75
*n* = 12, 10	1.00	4.42	4.96	3.67	4.19
	0.75	3.98	4.55	3.31	3.99
	0.50	3.88	4.15	3.22	3.72
	0.25	3.88	3.82	3.23	3.49
	0.00	4.49	3.23	3.70	2.91
*L. striatellus*	2.00	4.57	4.09	3.81	3.24
*n* = 9, 8	1.00	3.78	3.52	3.26	2.71
	0.75	3.65	3.22	3.15	2.59
	0.50	3.61	2.90	2.99	2.31
	0.25	3.64	2.89	3.03	2.28
	0.00	4.19	4.04	3.22	3.20

*n* is the sample size for the development period of each rice planthopper in each plot; the normal plot sample size is on the left, and the low-water temperature plot sample size is on the right.

## Discussion

### Influence of Water Temperature Differences on Canopy Air Temperature

Diurnal temperature variation in paddy fields is known to be smaller than that in upland fields due to the large volumetric heat capacity of water. In particular, the diurnal temperature variation is lower inside the canopy than outside the canopy. Diurnal temperature variation is known to affect pest development (Chen et al. 2015). It has been reported that the development rate of herbivorous arthropods decreases when the mean daily temperature is around 30 °C and the diurnal temperature variation is over 10 °C (Vangansbeke et al. 2015). In this study, diurnal temperature variation inside the canopy was lower than 10 °C even on days when the diurnal temperature variation outside the canopy was 10 °C or higher. Although the development of rice planthoppers depends on the effect of the diurnal temperature variation, it is possible that the environment inside the paddy field canopy functions as a buffer, thereby reducing the effect of the diurnal temperature variation on the accuracy of development prediction. Lowering the water temperature by continuous irrigation has been proven to be effective for improving rice quality and preventing sheath blight disease (Arai and Ito 2001, Nagata et al. 2005, Miyasaka et al. 2011). In this study, we established low-water temperature plots via continuous irrigation to collect broad-range temperature data inside the canopy. Continuous irrigation resulted in an average reduction of 2 °C in water temperature per day, consistent with the results of previous studies (Arai and Ito 2001, Miyasaka et al. 2011). However, no relationship was found between the observed development periods of the 3 rice planthopper species and the water treatment plots, nor was there a statistically significant difference. The 2 °C water temperature difference inside the canopy had an average effect of 0.8 °C on the air temperature at the most affected height (0.25 m). For rice planthoppers such as *N. lugens*, a temperature difference of 0.8 °C may result in a difference of 1–2 days in their development period of approximately 30 days. Given that the survey frequency in this study was 3–4 days, it may not have been possible to detect differences in the developmental period due to temperature differences between inside and outside the canopy. However, our results suggest that extending the development periods of rice planthopper by more than 3 days is difficult, even with continuous irrigation to lower the water temperature. Therefore, further investigation with more frequent observations or drastic changes in water temperature (>2 °C) is required to verify the effects of water temperature control on the development rate of rice planthoppers.

### Influence of Air Temperatures Inside the Canopy on Predicting Pest Developments

In this study, analyzing the development periods of the 3 rice planthopper species and air temperatures by height revealed that the predictions of the development period using air temperatures inside the canopy were in better agreement with the observed values compared to predictions using only the reference heights. In most instances, the development periods predicted using the air temperature at the reference height (2.0 m) were shorter than the actual observed periods. This discrepancy may be attributable to the air temperature at the reference height (2.0 m) being higher than that within the canopy, where the 3 rice planthopper species reside. The phenology model used in this study indicated that the development rate of *N. lugens* was the highest at temperatures ranging from 28.5 to 34.9 °C. The air temperature at the reference height (2.0 m) was often within this range compared to the temperature inside the canopy. Additionally, the phenology model did not show a decrease in development rate at higher temperatures, as the maximum temperature during the study was 34.6 °C below *T*_stop_ (34.9 °C). Consequently, the predicted development periods were likely shorter because the predicted development rates exceeded the actual rates. It is also possible that the observed development periods were extended owing to stunted development under high-temperature conditions in the fields. Indeed, there are reports of stunted development of and reduced egg-laying by rice planthoppers at 32 °C or higher (Park et al. 2011, 2013, Sujithra and Chander 2013, Wang et al. 2013, Vattikuti et al. 2019, Horgan et al. 2020). However, development period extensions (i.e., a decrease in the development rate) were not observed when *N. lugens* individuals were reared at 34–38 °C for a short time (11:00–16:00) instead of continuously at 32 °C (Yang et al. 2021). This finding suggests that the discrepancy between the predicted and observed development periods using air temperature at the reference height (2.0 m) is unlikely to be attributed to the omission of observing the reduced development rates between *T*_upper_ and *T*_stop_. Nighttime air temperatures inside the canopy dropped below *T*_min_ (12.7 °C) in late October, which was the water drainage time before paddy rice harvest. In most cases, temperatures at the reference height (2.0 m) also dropped below *T*_min_. Differences in air temperatures inside and outside the canopy may influence development rates when daytime air temperatures within the canopy drop below *T*_min_. However, temperatures below 15 °C are generally unsuitable for rice cultivation (Sánchez et al. 2014, Kuwagata et al. 2019). While data remain insufficient, considering air temperature differences inside and outside the canopy are rare when temperatures are near *T*_min_ in Asian paddy rice cultivation.

In this study, *N. lugens* showed the highest RMSE_ss_, an indicator of improvement in the RMSE, in both normal and low-water temperature plots. *Nilaparvata lugens* tends to inhabit the bottom of rice plants, whereas *S. furcifera* and *L. striatellus* have not been reported to prefer specific parts of rice plants at any developmental stages (e.g., eggs, nymphs, and adults) (Takai and Inou 1970, Noda 1987, Isichaikul and Ichikawa 1993, Matsumura 2017). Considering that there are differences of 0.7 °C in the normal plots and 1.1 °C in the low-water temperature plots between the bottom (0.25 m) and top (1.0 m), even within the same canopy, differences in the microhabitats of the three rice planthopper species may affect the RMSE_ss_. Thus, these results suggest the importance of using environmental information on the habitats of pests to predict their development periods. For all 3 species, the RMSE_ss_ values based on the prediction of the development period using the air temperature at the reference height (2.0 m) and 0.25 m inside the canopy were higher in the low-water temperature plots than in the normal plots. We observed that the lower the water temperature, the greater the difference between the air temperature at the reference height (2.0 m) and the bottom of the canopy (0.25 m), which is consistent with previous reports (Maruyama et al. 2023). These findings revealed that in paddy fields where the water temperature is low, predicting pest development using the air temperature inside the canopy (where pests live) would be more effective than using air temperature at the reference height. As it is often difficult to observe the weather in mountainous paddy fields, incorporating models that estimate the temperature inside a canopy using micrometeorological models along with regional atmospheric models such as WRF is necessary for accurate predictions of pest development (Maruyama and Kuwagata 2010, Chen et al. 2011, Yoshimoto et al. 2011, Skamarock et al. 2021).

The main limitation of this study was focusing solely on the effects of temperature on development period prediction for the 3 rice planthopper species. Furthermore, competition among species and other stress responses was not investigated. Predicted development periods in this study tended to be shorter than the observed period, even when considering air temperature within the canopy. Additionally, factors other than temperature may influence development periods (Rashid et al. 2016, Ikeuchi and Kubota 2018, Vailla et al. 2019, Liu et al. 2020). It has been established that feeding *S. furcifera* with paddy rice damaged by the same species slows their development rate (Matsumura and Suzuki 2003). In addition, *L. striatellus* is known to migrate to or from maize during the cultivation of paddy rice because *L. striatellus* hosts crops other than paddy rice (Yoshida et al. 2014). We believe that this migration is one factor that decreases the accuracy of on-site development prediction, not only for rice planthoppers but also for other pests. In *L. striatellus*, a species that has multiple host crops, the development prediction based on the temperature inside the canopy showed slightly improved accuracy; however, this effect was limited compared to that of the development prediction of *N. lugens*, which prefers to inhabit the lower sections of rice plants. The migration of pests may obscure population peaks, which are important in determining their development period. In addition, the migration of pests leads to changes in not only the canopy environment conditions, such as temperature, experienced by the pest but also in the development rate owing to changes in diet (Wang et al. 2013, HE et al. 2021). Therefore, depending on the type of pest, population density may need to be surveyed on surrounding crops other than the target crop. In the future, developing and modifying the model to consider these factors, such as the migration between crops and feeding conditions, will likely be the key to further improving prediction accuracy and reducing paddy rice damage caused by rice planthoppers.

Accurate pest development predictions are vital for optimizing labor utilization and ensuring a stable food supply in agriculture. In this study, we demonstrated the importance of considering the microclimate within a crop canopy in predicting crop growth and disease outbreaks, as well as developing pests. In particular, our results indicated that *N. lugens* may be strongly affected by microclimate inside the lower canopy, which represents a microhabitat within the rice canopy. These results highlight the importance of considering the specific temperature point at which data is recorded while acknowledging that other factors might be equally important in predicting pest development. We anticipate the utilization of microclimate-based predictions of pest development to serve as an effective means of bridging the gap between laboratory-developed phenology models and actual pest developments observed in the field.

## References

[CIT0001] Arai Y , Ito H. Effect of flow irrigation on high temperature ripening in paddy field rice. Tohoku J Crop Sci. 2001:44:89–90. 10.20725/tjcs.44.0_89

[CIT0002] Bergman TL , Lavine AS, Incropera FP, DeWitt DP. Fundamentals of heat and mass transfer. 8th ed. New York: John Wiley & Sons, Ltd; 2020. 10.1007/978-3-319-15793-1_19

[CIT0003] Bottrell DG , Schoenly KG. Resurrecting the ghost of green revolutions past: the brown planthopper as a recurring threat to high-yielding rice production in tropical Asia. J Asia-Pac Entomol. 2012:15(1):122–140. 10.1016/j.aspen.2011.09.004

[CIT0004] Chen F , Kusaka H, Bornstein R, Ching J, Grimmond CSB, Grossman-Clarke S, Loridan T, Manning KW, Martilli A, Miao S. The integrated WRF/urban modelling system: development, evaluation, and applications to urban environmental problems. Int J Climatol. 2011:31(2):273–288. 10.1002/joc.2158

[CIT0005] Chen S , Fleischer S, Saunders M, Thomas MB. The influence of diurnal temperature variation on degree-day accumulation and insect life history. PLoS One. 2015:10(3):1–15. 10.1371/journal.pone.0120772 PMC4366191 25790195

[CIT0006] Cheng JJ , Li H, Ren B, Zhou C-J, Kang Z-S, Huang L-L. Effect of canopy temperature on the stripe rust resistance of wheat. NZ J Crop Hort Sci. 2015:43(4):306–315. 10.1080/01140671.2015.1098708

[CIT0007] Damos P , Savopoulou-Soultani M. Temperature-driven models for insect development and vital thermal requirements. Psyche. 2012:2012:1–13. 10.1155/2012/123405

[CIT0008] Davidson J. On the relationship between temperature and rate of development of insects at constant temperatures. J Anim Ecol. 1944:13(1):26. 10.2307/1326

[CIT0009] Davidson NA , Wilson LT, Hoffmann MP, Zalom FG. Comparisons of temperature measurements from local weather stations and the tomato plant canopy: implications for crop and pest forecasting. J Am Soc Hortic Sci. 1990:115(5):861–869. 10.21273/jashs.115.5.861

[CIT0010] Faye E , Rebaudo F, Carpio C, Herrera M, Dangles O. Does heterogeneity in crop canopy microclimates matter for pests? Evidence from aerial high-resolution thermography. Agric Ecosyst Environ. 2017:246:124–133. 10.1016/j.agee.2017.05.027

[CIT0011] Fukuoka M , Yoshimoto M, Hasegawa T. Mincer: a novel instrument for monitoring the micrometeorology of rice canopies. J Agric Meteorol. 2012:68(2):135–147. 10.2480/agrmet.68.2.1

[CIT0012] Haider N , Kirkeby C, Kristensen B, Kjær LJ, Sørensen JH, Bødker R. Microclimatic temperatures increase the potential for vector-borne disease transmission in the Scandinavian climate. Sci Rep. 2017:7(1):8175. 10.1038/s41598-017-08514-9 28811576 PMC5557972

[CIT0013] Hatfield J. Biometeorology in integrated pest management. 1st ed. Cambridge (MA): Academic Press; 1982. 10.1016/B978-0-12-332850-2.X5001-4

[CIT0014] He L , Wang T, Chen Y, Ge SS, Wyckhuys KAG, Wu KM. Larval diet affects development and reproduction of East Asian strain of the fall armyworm, *Spodoptera frugiperda*. J Integr Agric. 2021:20(3):736–744. 10.1016/s2095-3119(19)62879-0

[CIT0015] Hilbert DW , Logan JA. Empirical model of nymphal development for the migratory grasshopper, *Melanoplus sanguinipes* (Orthoptera: Acrididae). Environ Entomol. 1983:12(1):1–5. 10.1093/ee/12.1.1

[CIT0016] Hirae M , Shiba T. Forecasting methods of the occurrence in the small brown planthopper by using yellow sticky trap and the effective cumulative temperature calculation of the JPP-NET. *Plant protection.* 2016:70(2):79–83.

[CIT0017] Honek A , Kocourek F. Temperature and development time in insects: a general relationship between thermal constants. Zool Jahrb Abt Syst Okol Geogr Tiere. 1990:117:401–439.

[CIT0018] Horgan FG , Arida A, Ardestani G, Almazan MLP. Temperature-dependent oviposition and nymph performance reveal distinct thermal niches of coexisting planthoppers with similar thresholds for development. PLoS One. 2020:15(6):e0235506. 10.1371/journal.pone.0235506 32603337 PMC7326231

[CIT0019] Horgan FG , Ramal AF, Bentur JS, Kumar R, Bhanu KV, Sarao PS, Iswanto EH, Van Chien H, Phyu MH, Bernal CC, et al. Virulence of brown planthopper (*Nilaparvata lugens*) populations from South and South East Asia against resistant rice varieties. Crop Prot. 2015:78:222–231. 10.1016/j.cropro.2015.09.014

[CIT0020] Ikeuchi S , Kubota S. The effect of mid-summer drainage days at the organic paddy field on population growth of the brown planthopper (*Nilaparvata lugens* Stal). Bull Ehime Res Inst Agric Fish. 2018:10:33–39.

[CIT0021] Isichaikul S , Ichikawa T. Relative humidity as an environmental factor determining the microhabitat of the nymphs of the rice brown planthopper, *Nilaparvata lugens* (Stål) (Homoptera: Delphacidae). Popul Ecol. 1993:35(2):361–373. 10.1007/bf02513607

[CIT0022] Jiang YD , Yuan X, Bai YL, Wang G-Y, Zhou W-W, Zhu Z-R. Knockdown of timeless disrupts the circadian behavioral rhythms in *Laodelphax striatellus* (Hemiptera: Delphacidae). Environ Entomol. 2018:47(5):1216–1225. 10.1093/ee/nvy095 30059997

[CIT0023] Kiritani K. The low development threshold temperature and the thermal constant in insects and mites in Japan. Bull Natl Inst Agro-Environ Sci. 2012:31:1–74. 10.24514/00002995

[CIT0024] Kisimoto R. Synoptic weather conditions inducing long‐distance immigration of planthoppers, *Sogatella furcifera* Horvath and *Nilaparvata lugens* Stal. Ecol Entomol. 1976:1(2):95–109. 10.1111/j.1365-2311.1976.tb01210.x

[CIT0025] Kontodimas DC , Eliopoulos PA, Stathas GJ, Economou LP. Comparative temperature-dependent development of *Nephus includens* (Kirsch) and *Nephus bisignatus* (Boheman) (Coleoptera: Coccinellidae) preying on *Planococcus citri* (Risso) (Homoptera: Pseudococcidae): evaluation of a linear and various nonlinear models using specific criteria. Environ Entomol. 2004:33(1):1–11. 10.1603/0046-225x-33.1.1

[CIT0026] Krishnaiah NV , Prasad R, Rao R, Pasalu IC, Lakshmi J, Narayana L, Lingaiah T. Effect of constant and variable temperatures on biological parameters of rice brown planthopper, *Nilaparvata lugens* (Stal). Indian J Plant Prot. 2005:33:181–187.

[CIT0027] Kuno E. Studies on the population dynamics of rice leafhoppers in a paddy field. Bull Kyushu Agric Exp Stn. 1968:14:131–246.

[CIT0028] Kuwagata T , Ikawa H, Maruyama A, Ono K, Yoshimoto M, Ishida S, Watanabe T. Micro-climate model for a rice paddy field and its application to agriculture. Low Temp Sci. 2019:77:125–136. 10.14943/lowtemsci.77.125

[CIT0029] Lee JH , Choi JY, Tao XY, Kim JS, Kim W, Je YH. Transcriptome analysis of the small brown planthopper, *Laodelphax striatellus* carrying rice stripe virus. Plant Pathol J. 2013:29(3):330–337. 10.5423/PPJ.NT.01.2013.0001 25288960 PMC4174806

[CIT0030] Li JM , Zhou YR, Sun ZT, Wang X, Xie L, Chen J-P. Identification and profiling of conserved and novel microRNAs in *Laodelphax striatellus* in response to rice black-streaked dwarf virus (RBSDV) infection. Genomics Data. 2015:3:63–69. 10.1016/j.gdata.2014.08.010 26484150 PMC4536020

[CIT0031] Liu J , Zhuang J, Huang W, Chi H, Wang C, Hua H, Wu G. Different adaptability of the brown planthopper, *Nilaparvata lugens* (Stål), to gradual and abrupt increases in atmospheric CO_2_. J Pest Sci. 2020:93(3):979–991. 10.1007/s10340-020-01221-x

[CIT0032] Maruyama A , Kuwagata T. Coupling land surface and crop growth models to estimate the effects of changes in the growing season on energy balance and water use of rice paddies. Agric For Meteorol. 2010:150(7–8):919–930. 10.1016/j.agrformet.2010.02.011

[CIT0033] Maruyama A , Kuwagata T, Watanabe T. Observations on dew formation in the rice canopy and its simulation using a multilayer microclimate model. J Agric Meteorol. 2023:79(1):28–37. 10.2480/agrmet.d-22-00016

[CIT0034] Maruyama A , Matsumoto Y, Nakagawa H. Multiple-globe thermometer for measuring the air temperature without an aspirated radiation shield. Agric For Meteorol. 2020:292-293(1):108028. 10.1016/j.agrformet.2020.108028

[CIT0035] Matsumura M. Planthopper protection handbook. Tokyo (Japan): Nobunkyo; 2017. p. 96.

[CIT0036] Matsumura M , Suzuki Y. Direct and feeding-induced interactions between two rice planthoppers, *Sogatella furcifera* and *Nilaparvata lugens*: effects on dispersal capability and performance. Ecol Entomol. 2003:28(2):174–182. 10.1046/j.1365-2311.2003.00498.x

[CIT0037] Miyasaka A , Nakajima T, Maruyama A, Wakiyama Y. Effects of lower water temperature, by water streaming treatment during ripening stage, on sheath blight disease and white immature kernels in rice. Kyushu Plant Prot Res. 2011:57:1–6. 10.4241/KYUBYOCHU.57.1

[CIT0038] Nagata K , Kodani T, Yoshinaga S, Fukuda A. Effects of water managements during the early grain-filling stage on grain fissuring in rice. Tohoku J Crop Sci. 2005:48:33–35. 10.20725/TJCS.48.0_33

[CIT0039] Nagata T , Masuda T. Efficiency of sticky boards for population estimation of the brown planthopper, *Nilaparvata lugens* (STAL) (Hemiptera: Delphasidae) on rice hills. Appl Entomol Zool. 1978:13(2):55–62. 10.1303/aez.13.55

[CIT0040] Nietschke BS , Magarey RD, Borchert DM, Calvin DD, Jones E. A developmental database to support insect phenology models. Crop Prot. 2007:26(9):1444–1448. 10.1016/j.cropro.2006.12.006

[CIT0041] Noda H. Vertical distribution and position of rice planthoppers on the host rice plant (Homoptera: Delphacidae). Jpn J Appl Entomol Zool. 1987:31(2):156–161. 10.1303/jjaez.31.156

[CIT0042] Noda H. Developmental zero and total effective temperature of three rice planthoppers (Homoptera: Delphacidae). Jpn J Appl Entomol Zool. 1989:33(4):263–266. 10.1303/jjaez.33.263

[CIT0043] Okuda M , Hirae M, Shiba T, Suwa N, Shimizu M. Prediction of the emergence date of small brown planthopper (*Laodelphax striatellus*) by the effective cumulative temperature method and the agro-meteorological grid square data system. Annu Rep Kanto-Tosan Plant Prot Soc. 2019:66:52–55. 10.11337/ktpps.2019.

[CIT0044] Otuka A. Migration of rice planthoppers and their vectored re-emerging and novel rice viruses in East Asia. Front Microbiol. 2013:4:309. 10.3389/fmicb.2013.00309 24312081 PMC3836001

[CIT0045] Pangga IB , Hanan J, Chakraborty S. Climate change impacts on plant canopy architecture: implications for pest and pathogen management. Eur J Plant Pathol. 2013:135(3):595–610. 10.1007/s10658-012-0118-y

[CIT0046] Park C-G , Kim K-H, Park H-H, Lee S-G. Temperature-dependent development model of white backed planthopper (WBPH), *Sogatella furcifera* (Horvath) (Homoptera: Delphacidae). Korean J Appl Entomol. 2013:52(2):133–140. 10.5656/ksae.2013.02.1.070

[CIT0047] Park C-G , Park H-H, Kim K-H. Temperature-dependent development model and forecasting of adult emergence of overwintered small brown planthopper, *Laodelphax striatellus* Fallen, population. Korean J Appl Entomol. 2011:50(4):343–352. 10.5656/ksae.2011.10.0.62

[CIT0048] Piyaphongkul J , Pritchard J, Bale J. Can tropical insects stand the heat? A case study with the brown planthopper *Nilaparvata lugens* (Stål). PLoS One. 2012:7(1):e29409. 10.1371/journal.pone.0029409 22253720 PMC3257224

[CIT0049] R Core Team. R: a language and environment for statistical computing. Vienna (Austria): R Foundation for Statistical Computing; 2021. https://www.R-project.org.

[CIT0050] Rashid MM , Jahan M, Islam KS. Impact of nitrogen, phosphorus and potassium on brown planthopper and tolerance of its host rice plants. Rice Sci. 2016:23(3):119–131. 10.1016/j.rsci.2016.04.001

[CIT0051] Rebaudo F , Faye E, Dangles O. Microclimate data improve predictions of insect abundance models based on calibrated spatiotemporal temperatures. Front Physiol. 2016:7:139. 10.3389/fphys.2016.00139 27148077 PMC4836147

[CIT0052] Sánchez B , Rasmussen A, Porter JR. Temperatures and the growth and development of maize and rice: a review. Glob Chang Biol. 2014:20(2):408–417. 10.1111/gcb.12389 24038930

[CIT0053] Saudreau M , Pincebourde S, Dassot M, Adam B, Loxdale HD, Biron DG. On the canopy structure manipulation to buffer climate change effects on insect herbivore development. Trees. 2013:27(1):239–248. 10.1007/s00468-012-0791-7

[CIT0054] Shiba T , Hirae M, Hayano-Saito Y, Ohto Y, Uematsu H, Sugiyama A, Okuda M. Spread and yield loss mechanisms of rice stripe disease in rice paddies. Field Crops Res. 2018:217:211–217. 10.1016/j.fcr.2017.12.002

[CIT0055] Skamarock WC , Klemp JB, Dudhia J, Gill DO, Liu Z, Berner J, Wang W, Powers JG, Duda MG, Barker DM, et al. *A Description of the Advanced Research WRF Model Version 4.3.* National Center for Atmospheric Research. Boulder, CO (USA). 2021:TN-556+STR. 10.5065/1dfh-6p97

[CIT0056] Suenaga H. Analytical studies on the ecology of two species of planthoppers, the whitebacked planthopper (*Sogata furcifera* Horváth) and the brown planthopper (*Nilaparvata lugens* Stål), with special reference to their outbreaks. Bull Kyushu Agric Exp Stn. 1963:8(1):1–152.

[CIT0057] Sujithra M , Chander S. Simulation of rice brown planthopper, *Nilaparvata lugens* (Stal) population and crop-pest interactions to assess climate change impact. Clim Change. 2013:121(2):331–347. 10.1007/s10584-013-0878-1

[CIT0058] Sun Y , Wu Y, Sun Y, Luo Y, Guo C, Li B, Li F, Xing M, Yang Z, Ma J. 2022 Effects of water and nitrogen on grain filling characteristics, canopy microclimate with chalkiness of directly seeded rice. Agriculture. 2022:12(1):122. 10.3390/agriculture12010122

[CIT0059] Takai A , Inou M. Comparison of distribution concentration between white backed planthopper and brown planthopper. Annu Rep Ibaraki Plant Prot Soc. 1970:10:22–23.

[CIT0060] Takai T , Yano M, Yamamoto T. Canopy temperature on clear and cloudy days can be used to estimate varietal differences in stomatal conductance in rice. Field Crops Res. 2010:115(2):165–170. 10.1016/j.fcr.2009.10.019

[CIT0061] Tanaka K , Otuka A, Matsumura M. Generation forecast of *Nilaparvata lugens* using forecast data of agro-meteorological grid square data system. Kyushu Plant Prot Res. 2019:65:75–83. 10.4241/kyubyochu.65.75

[CIT0062] Tonnang HEZ , Hervé BDB, Biber-Freudenberger L, Salifu D, Subramanian S, Ngowi VB, Guimapi RYA, Anani B, Kakmeni FMM, Affognon H. Advances in crop insect modelling methods—towards a whole system approach. Ecol Modell. 2017:354:88–103. 10.1016/j.ecolmodel.2017.03.015

[CIT0063] Tsujimoto Y , Fuseini A, Inusah BIY, Dogbe W, Yoshimoto M, Fukuoka M. Different effects of water-saving management on canopy microclimate, spikelet sterility, and rice yield in the dry and wet seasons of the sub-humid tropics in northern Ghana. Field Crops Res. 2021:260:107978. 10.1016/j.fcr.2020.107978

[CIT0064] Utida S. Developmental zero temperature in insect. Jpn J Appl Entomol Zool. 1957:1(1):46–53. 10.1303/jjaez.1.46

[CIT0065] Uvarov BP. Insects and climate. Trans R Entomol Soc Lond. 1931:79(1):1–232. 10.1111/j.1365-2311.1931.tb00696.x

[CIT0066] Vailla S , Muthusamy S, Konijeti C, Shanker C, Vattikuti JL. Effects of elevated carbon dioxide and temperature on rice brown planthopper, *Nilaparvata lugens* (Stål) populations in India. Curr Sci. 2019:116(6):988–996. 10.18520/cs/v116/i6/988-996

[CIT0067] Vangansbeke D , Audenaert J, Nguyen D, Verhoeven R, Gobin B, Tirry L, De Clercq P. Diurnal temperature variations affect development of a herbivorous arthropod pest and its predators. PLoS One. 2015:10:1–19. 10.1371/journal.pone.0124898 PMC4398551 25874697

[CIT0068] Vattikuti J , Sailaja V, Prasad YG, Katti GR, Chirutkar PM, Rao GR, Padmakumari AP, Prabhakar CH, Padmavathi M. Temperature driven development of the rice brown planthopper, *Nilaparvata lugens* (Stål) (Hemiptera: Delphacidae). J Agrometeorol. 2019:21:131–140. 10.54386/jam.v21i2.221

[CIT0069] Wang L , Shi P, Chen C, Xue F. Effect of temperature on the development of *Laodelphax striatellus* (Homoptera: Delphacidae). J Econ Entomol. 2013:106(1):107–114. 10.1603/ec12364 23448021

[CIT0070] Yang L , Huang LF, Wang WL, Chen E-H, Chen H-S, Jiang J-J. Effects of temperature on growth and development of the brown planthopper, *Nilaparvata lugens* (Homoptera: Delphacidae). Environ Entomol. 2021:50(1):1–11. 10.1093/ee/nvaa144 33205198

[CIT0071] Yoshida K , Matsukura K, Sakai J, Onuki M, Sanada-Morimura S, Towata T, Matsumura M. Seasonal occurrence of *Laodelphax striatellus* (Hemiptera: Delphacidae) in a rice-forage crops mixed cropping area in central Kyushu, Japan. Appl Entomol Zool. 2014:49(3):475–481. 10.1007/s13355-014-0275-x

[CIT0072] Yoshimoto M , Fukuoka M, Hasegawa T, Utsumi M, Ishigooka Y, Kuwagata T. Integrated micrometeorology model for panicle and canopy temperature (IM2PACT) for rice heat stress studies under climate change. J Agric Meteorol. 2011:67(4):233–247. 10.2480/agrmet.67.4.8

[CIT0073] Yoshimoto M , Fukuoka M, Tsujimoto Y, Matsui T, Kobayasi K, Saito K, van Oort PAJ, Inusah BIY, Vijayalakshmi C, Vijayalakshmi D, et al. Monitoring canopy micrometeorology in diverse climates to improve the prediction of heat-induced spikelet sterility in rice under climate change. Agric For Meteorol. 2022:316:108860. 10.1016/j.agrformet.2022.108860

